# Concurrent sigmoid colon adenocarcinoma presenting with cutaneous adult T-cell leukemia/lymphoma: a rare case report

**DOI:** 10.1186/s13027-026-00755-6

**Published:** 2026-04-11

**Authors:** Amirhosein Maharati, Alireza Zangooie, Maryam Sardarkhani Eydgahi, Hoda Haghshenas, Zahra Eghbali, Naser Tayyebi Meibodi, Zahra Salehi, Abolghasem Allahyari

**Affiliations:** 1 Endoscopic and Minimally Invasive Surgery Research Center, Mashhad University of Medical Sciences, Mashhad, Iran; 2 Cell Therapy and Hematopoietic Stem Cell Transplantation Research Center, Research Institute for Oncology, Hematology and Cell Therapy, Tehran University of Medical Sciences, Tehran, Iran; 3 Hematology, Oncology and Stem Cell Transplantation Research Center, Research Institute for Oncology, Hematology and Cell Therapy, Tehran University of Medical Sciences, Tehran, Iran; 4 Department of Pathology, Faculty of Medicine, Mashhad University of Medical Sciences, Mashhad, Iran; 5 Faculty of Medicine, Jahrom University of Medical Sciences (JUMS), Jahrom, Iran; 6 Cutaneous Leishmaniasis Research Center, Imam Reza Hospital, Mashhad University of Medical Sciences, Mashhad, Iran; 7 Division of Hematology and Oncology, Department of Internal Medicine, Faculty of Medicine, Mashhad University of Medical Sciences, Mashhad, Iran

**Keywords:** Adult T-cell leukemia/lymphoma, Cutaneous ATLL, Sigmoid colon adenocarcinoma, HTLV-1, Case report

## Abstract

**Background:**

Adult T-cell leukemia/lymphoma (ATLL) is a rare peripheral T-cell malignancy induced by Human T-cell lymphotropic virus type 1 (HTLV-1) infection, commonly affecting individuals in their 50s to 60s. The disease manifests through various clinical forms, with skin involvement being a frequent presentation. While HTLV-1 has been linked to several cancers, including non-ATLL lymphomas and liver cancer. This case report discusses the rare coexistence of sigmoid colon adenocarcinoma and cutaneous ATLL in a 75-year-old woman.

**Materials and methods:**

A 75-year-old woman presenting with abdominal pain, vomiting, distention, and constipation underwent clinical evaluation including physical examination, laboratory tests, abdominal imaging, and contrast-enhanced CT. Due to large-bowel obstruction, she received emergency surgical management consisting of left hemicolectomy, splenic flexure mobilization, regional lymphadenectomy, and Hartmann colostomy. Tissue from the resected colon and later cutaneous lesions was submitted for histopathological examination and immunophenotypic profiling with an extended T-cell marker panel. Serologic testing for HTLV-1 was also performed. Additionally, a systematic literature review was conducted according to PRISMA 2020 guidelines. Comprehensive searches of PubMed, Scopus, and Web of Science from database inception to November 3, 2025, used controlled vocabulary and free-text terms related to HTLV-1/ATLL and gastrointestinal malignancies. Case reports and case series involving human subjects were independently screened in two phases.

**Results:**

The patient was diagnosed with sigmoid colon adenocarcinoma requiring emergency surgery and later developed cutaneous lesions, which were confirmed as smoldering ATLL through histopathology, immunophenotyping (mature CD4^+^/CD25^+^ T-cell phenotype with CD7 loss), and positive HTLV-1 serology. She is receiving adjuvant chemotherapy for colorectal cancer while undergoing surveillance for ATLL. The systematic search identified 365 records; after removal of duplicates and screening, four eligible publications were included. Of these four studies, three were case reports, all describing patients with HTLV-1 infection and gastric adenocarcinoma. The fourth publication was a case series including three patients; two of them ATLL and gastric adenocarcinoma, consistent with the findings of our study, while the third patient had ATLL and colorectal adenocarcinoma. Overall, the reported clinical outcomes varied, underscoring the rarity of this coexistence and the lack of standardized management approaches.

**Conclusion:**

This case highlights the rare occurrence of sigmoid colon adenocarcinoma and cutaneous ATLL in an HTLV-1-positive patient, emphasizing the potential for HTLV-1 to contribute to multiple malignancies. Our findings underscore the need for further research to explore the link between HTLV1 and gastrointestinal cancers, as well as its broader oncogenic potential in other malignancies. The coexistence of these two diseases presents diagnostic challenges and calls for an integrated approach to patient management in HTLV-1-endemic areas.

**Supplementary information:**

The online version contains supplementary material available at 10.1186/s13027-026-00755-6.

## Introduction

Adult T-cell leukemia/lymphoma (ATLL) is a mature peripheral T-cell malignancy caused by lifelong infection with HTLV-1, a virus transmitted through breastfeeding, blood transfusion, shared needles, and sexual contact. Only about 4–7% of infected individuals eventually develop ATLL, typically in their 50s to 60s [1, 2]. Based on Shimoyama criteria, ATLL is classified into four subtypes, including acute, lymphomatous, chronic, and smoldering. The aggressive acute and lymphomatous forms are most common, often presenting with lymphadenopathy, hepatosplenomegaly, hypercalcemia, high LDH (in the acute type), and widespread organ involvement, particularly of the skin, lungs, bones, and liver; the lymphomatous type usually has minimal circulating ATLL cells. In contrast, the chronic and smoldering forms show a lower disease burden, distinct patterns of lymphocytosis and LDH levels, and more limited involvement confined to the skin, lungs, or liver, without hypercalcemia [2].

Skin involvement is common in ATLL, affecting nearly half of patients and in some cases serving as the initial clinical sign. Cutaneous manifestations vary widely in appearance, and certain patterns of eruption can provide prognostic information. In addition to disease-specific lesions, patients may develop nonspecific skin changes and are prone to cutaneous and systemic infections. Because these lesions display substantial clinical and histopathologic variability, distinguishing ATLL from other dermatologic conditions can be difficult [3]. Early and accurate recognition of ATLL-related skin findings is therefore essential for timely diagnosis and appropriate treatment [4].

Previous studies have confirmed that HTLV-1 infection directly contributes to the development of ATLL. Additional research has indicated that HTLV-1 may also play a role in the development of other cancers, including non-ATL lymphomas, liver cancer, and cervical cancer. However, these conclusions are primarily based on cross-sectional and case-control studies, which are susceptible to bias and methodological limitations [5–7]. To date, no cases of co-occurrence between ATLL and colorectal cancer have been reported, and no mechanisms linking HTLV-1 infection to synchronous gastrointestinal malignancies have been identified.

**Fig. 1:**
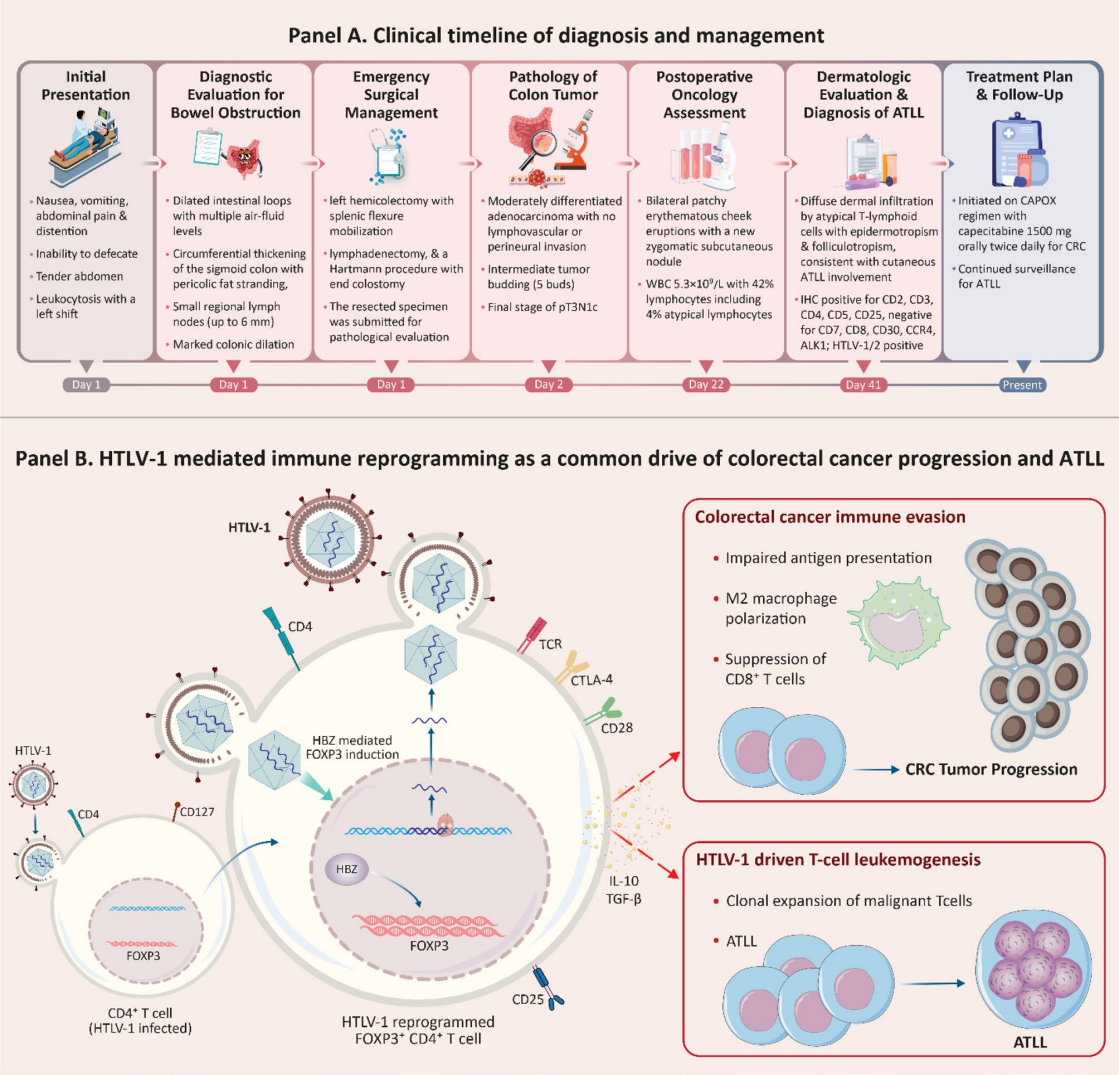
Chronological overview of clinical events and immunopathogenic mechanism for the concurrent presentation of sigmoid colon adenocarcinoma and cutaneous adult T-Cell Leukemia/Lymphoma (ATLL) in an HTLV-1 carrier. (**A**) We report a rare case of concurrent HTLV-1–associated ATLL and sigmoid colon cancer in a 75-year-old woman. The patient initially presented with abdominal symptoms, and imaging revealed bowel obstruction and sigmoid colon thickening, leading to emergency surgery. Pathology confirmed moderately differentiated adenocarcinoma (pT3n1c). Later, the patient developed skin eruptions, and a skin biopsy revealed smoldering ATLL. The patient is currently receiving chemotherapy for colon cancer and is under surveillance for ATLL. (**B**) HTLV-1 infection of CD4^+^/CD25^+^/FOXP3^+^ T cells induce expression of HBZ and Tax, promoting expansion of regulatory T cells and upregulation of IL-10. Elevated IL-10 suppresses dendritic cell maturation and cytotoxic T-cell activity while enhancing Treg and M2 macrophage development, generating a systemic immunosuppressive environment. This permissive immune state facilitates immune escape and progression of colorectal adenocarcinoma in the sigmoid colon. In parallel, HTLV-1–infected T cells undergo clonal evolution and malignant transformation, leading to cutaneous adult T-cell leukemia/lymphoma (ATLL). Thus, a shared axis of HTLV-1–driven Treg polarization, IL-10–mediated suppression, and impaired antitumor immunity provides a unified explanation for the simultaneous manifestation of CRC and ATLL

Here, we report a rare case of concurrent HTLV-1–associated ATLL and sigmoid colon cancer in a 75-year-old woman (Fig. 1). To better understand this unusual coexistence, we also conducted a systematic review of the literature to identify previously reported cases of simultaneous ATLL and gastrointestinal malignancies in HTLV-1–positive individuals. This report highlights the clinical features, diagnostic challenges, and outcomes of patients with dual malignancies, providing insight into the broader oncogenic potential of HTLV-1 beyond ATLL.

## Materials and methods

### Patient clinical assessment

A 75-year-old woman presented to the emergency department with a 5-day history of nausea, vomiting, abdominal pain, abdominal distention, and absence of passage of flatus and stool; physical examination revealed a distended and tender abdomen. Initial evaluation included a complete physical examination, baseline blood tests, and plain abdominal radiography, followed by contrast-enhanced computed tomography of the abdomen and pelvis to further assess suspected bowel obstruction. Based on the clinical and radiologic findings, the patient underwent emergency laparotomy with left hemicolectomy, mobilization of the splenic flexure, regional lymphadenectomy, and a Hartmann procedure with end colostomy, and the resected specimen was submitted for pathological examination. Postoperatively, systemic workup included chest computed tomography and additional laboratory tests, including tumor markers and molecular assays for microsatellite instability. In view of the patient’s complaints of erythematous rashes on both cheeks and a subcutaneous nodule in the left zygomatic region, a complete dermatologic examination was performed, and a punch biopsy was obtained from the suspicious skin lesion; the biopsy specimen was sent for histopathologic and immunohistochemical analysis, and serum samples were collected for HTLV-1/2 serologic testing along with a peripheral blood smear to evaluate for atypical lymphocytes.

### Systematic literature screening

The entire process of study selection and data extraction was carried out in alignment with the PRISMA 2020 registration, ensuring a systematic and comprehensive assessment of the available literature. The PICO (Population, Intervention, Comparator, and Outcomes) framework for this review was structured as follows:

- ***Population:*** patients diagnosed with Human T-lymphotropic virus type 1 (HTLV-1) infection who concurrently presented with gastrointestinal malignancies.
- ***Intervention/Exposure:*** documented HTLV-1 infection or Adult T-cell Leukemia/Lymphoma (ATLL) associated with, involving, or coexisting with gastrointestinal cancers.
- ***Comparator:*** not applicable, as the included evidence primarily consisted of descriptive case reports and case series without control groups.
- ***Outcomes:*** clinical presentation, diagnostic findings, pathological and immunophenotypic characteristics, treatment approaches, response to therapy, and reported clinical outcomes.

#### Eligibility criteria for inclusion and exclusion

Studies were eligible for inclusion if they were case reports or case series describing patients diagnosed with Human T-lymphotropic virus type 1 (HTLV-1) infection in association with gastrointestinal malignancies.

To be included, reports were required to provide: (i) human subjects only; (ii) diagnostic or histopathologic confirmation of both HTLV-1 infection (or ATLL) and the gastrointestinal cancer; and (iii) sufficient clinical, laboratory, imaging, or pathological details to allow extraction of meaningful clinical information. Exclusion criteria consisted of the examination of non-human subjects (including animal models and/or commercial human or animal cell lines), review papers (literature reviews, systematic reviews, and meta-analyses), and non-English language papers. Studies for which full-text access remained unavailable, despite attempts to contact the authors, were also excluded. Additionally, there were no restrictions based on publication year, allowing inclusion of relevant studies from inception to the present.

#### Literature search

The literature search utilized three comprehensive databases: PubMed, SCOPUS, and Web of Science. PubMed queries incorporated relevant MeSH terms, while SCOPUS and Web of Science using predefined keywords tailored to capture three major categories of interest: “Human T lymphotropic virus type 1”, “Adult T-Cell Leukemia-Lymphoma” and “Gastrointestinal Neoplasms”. The search included studies published up to November 3, 2025, without language restrictions. The search strategy combined controlled vocabulary terms (e.g., MeSH, subject headings) with free-text keywords to maximize sensitivity. Terms encompassed disease entities, study designs, and descriptors of simultaneous occurrence (including *concurrent, coexisting, simultaneous, combined,* and *synchronous*) (see Supplementary File 1 for the complete strategy).

#### Screening and data extraction

All identified records were first imported into EndNote 21, where duplicate entries were automatically removed. The remaining references were then uploaded into the Rayyan screening platform, developed by the Qatar Computing Research Institute (QCRI) (http://www.rayyan.ai), to facilitate blinded and independent screening.

Two researchers (HH and AZ) conducted a two-phase screening process. In the initial stage, studies published as preprints or written in languages other than English were excluded. Titles and abstracts of the remaining records were evaluated to identify reports describing patients with HTLV-1 infection in association with gastrointestinal malignancies, with the aim of assessing their potential clinical and pathological interaction. Full texts of all potentially relevant articles were subsequently reviewed against predefined inclusion criteria. At each screening step (title/abstract and full text), the two reviewers compared decisions. Any disagreements were resolved through discussion, and if necessary, a third expert reviewer (ZS) provided an independent judgment.

For all studies that met the final inclusion criteria, the reviewers independently extracted detailed information on: publication year, study type, patient demographics (age, sex, geographic origin), relevant medical history and risk factors, clinical presentation, gastrointestinal involvement and other organ involvement, laboratory results, imaging and endoscopic findings, histopathology, immunophenotypic profile (including CD markers), HTLV-1 diagnostic method and viral status, molecular findings, treatment modalities (chemotherapy, surgery, supportive therapy), treatment response, and clinical outcomes.

#### Quality assessment and risk of bias

The quality of the included studies was assessed using the Joanna Briggs Institute (JBI) critical appraisal tools for case reports and case series [8]. These tools assess the methodological quality across eight domains for case reports and ten domains for case series. Quality assessment was performed independently by two reviewers, and disagreements were resolved by discussion.

## Results

### Case presentation

A 75-year-old woman from Neyshabour, Iran, with no significant past medical, drug, or family history presented to the emergency department with a five-day history of nausea, vomiting, abdominal pain, abdominal distention, and inability to defecate. On physical examination her abdomen was distended and tender. Initial laboratory testing demonstrated leukocytosis with a left shift (WBC 14.5 × 10^9/L; neutrophils 89%, lymphocytes 8%).

Work-up for bowel obstruction was initiated. Upright abdominal radiographs showed dilated intestinal loops with an increased gas pattern and multiple air-fluid levels. Contrast abdominal CT demonstrated circumferential thickening of the sigmoid colon (maximum wall thickness 13 mm) with pericolic fat stranding, regional lymphadenopathy (short axis diameter up to 6 mm), and marked dilation of the distal colon with a maximum diameter of 60 mm in the transverse colon and 73 mm in the cecum there was no findings in favor of liver metastasis. Findings were consistent with large-bowel obstruction secondary to a sigmoid mass. No focal hepatic lesions were identified. Biliary sludge (cumulative diameter 25 mm) and mild bilateral pleural effusions were noted; otherwise, there were no other significant intra-abdominal findings.

**Fig. 2:**
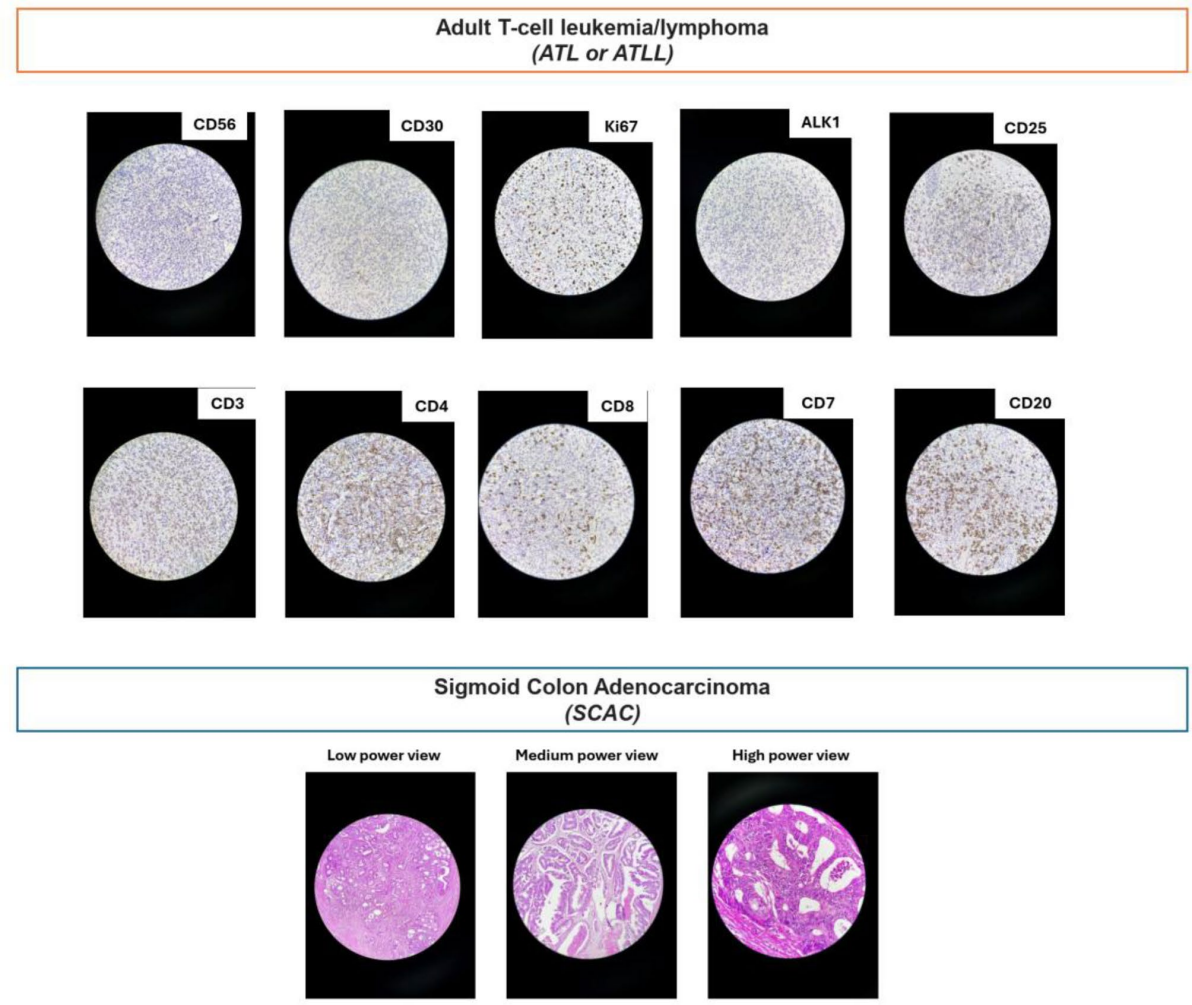
Histopathologic and immunophenotypic features of the patient’s concurrent sigmoid colon adenocarcinoma and cutaneous adult T-cell leukemia/lymphoma (ATLL). (**A**) Histopathologic and immunophenotypic features of the cutaneous ATLL lesion. The skin biopsy shows diffuse dermal infiltration by atypical lymphoid cells with epidermotropism and folliculotropism (H&E). Immunohistochemistry demonstrates a mature T-cell phenotype with strong expression of CD2, CD3, CD4, CD5 and CD25, and a high proliferative index by Ki-67. Loss of CD7 and absence of CD8, CD20, CD30, CD56, and ALK-1 expression support the diagnosis of ATLL. (**B**) Low- and high-power views of the sigmoid colon adenocarcinoma. Sigmoid biopsy shows atypical columnar cells with hyperchromatic, pleomorphic nuclei and crowded back-to-back glands. The tumor exhibits infiltrative and irregular glandular structures, with focal luminal necrosis

The patient was taken emergently to the operating room, where a left hemicolectomy with splenic flexure mobilization and regional lymphadenectomy was performed. No evidence of peritoneal carcinomatosis was observed intraoperatively. A Hartmann procedure with end colostomy was constructed. The resected specimen was submitted for histopathologic evaluation (Fig. 2 and Supplementary file 2).

The pathology department received a 14-cm segment of descending colon measuring 2.5 cm in diameter. A firm, solid mass was identified 4 cm from the distal resection margin. Histologic examination revealed a moderately differentiated adenocarcinoma measuring 7 × 3 × 1 cm, located within the descending colon. The tumor invaded through the muscularis propria into the pericolic adipose tissue without evidence of perforation, consistent with pT3 disease. No lymphatic, perineural, or lymphovascular invasion was identified. Tumor budding was categorized as intermediate, with five buds observed. Resection margins were negative. Ten regional lymph nodes were examined, all of which were free of metastasis. However, discrete tumor deposits were present within the pericolic soft tissue, corresponding to N1c classification

Postoperatively the patient was referred to our hematology-oncology clinic for adjuvant management. On review of systems, she reported bilateral, patchy, erythematous eruptions on both cheeks for six months, and a new erythematous, firm 2 × 1 cm subcutaneous nodule over the left zygomatic bone that had appeared one month prior to presentation. For metastatic work-up she underwent thoracic CT, IHC testing, and laboratory evaluation. Chest CT showed small bilateral reactive pleural effusions and three pulmonary nodules (right upper lobe 4 mm, right middle lobe 15 mm, left lower lobe 6 mm); the nodules lacked typical features of metastases and were judged not amenable to percutaneous biopsy by interventional radiology. Immunohistochemistry for mismatch-repair proteins demonstrated intact MLH1, MSH2, MSH6, and PMS2; this indicated the tumor was classified as mismatch repair-proficient (pMMR). Tumor markers were CEA = 1 ng/mL and CA-19-9 = 8.13 U/mL. Additional laboratory testing indicated vitamin D deficiency and positive hepatitis B core antibody (HBcAb).

Postoperative complete blood count demonstrated a WBC of 5.3 × 10^9^/L with the following differential: neutrophils 47%, lymphocytes 42% (including 5% atypical lymphocytes on peripheral blood smear), monocytes 9%, and eosinophils 2%. Given the presence of cutaneous lesions, the differential diagnosis included cutaneous metastasis, cutaneous lymphoma or pseudolymphoma, and other inflammatory or neoplastic dermatologic conditions. A punch biopsy of the zygomatic lesion was performed. Histopathologic examination revealed a diffuse dermal infiltrate of atypical T-lymphoid cells with epidermotropism and folliculotropism, findings consistent with cutaneous involvement by adult T-cell leukemia/lymphoma (ATLL). A comprehensive immunohistochemistry panel was performed (Fig. 2), and the results are summarized in Supplementary File 2. Serologic testing for HTLV-1/2 antibodies was positive (reported value: 12). In the context of positive HTLV serology, fewer than 4 × 10^9^/L circulating lymphocytes, and 5% atypical T lymphocytes in peripheral blood, the overall clinicopathologic picture supported a diagnosis of smoldering-type ATLL with cutaneous presentation.

For the patient’s stage II colon adenocarcinoma, adjuvant chemotherapy with oral capecitabine 1500 mg three times daily was initiated. Because of potential drug interactions between capecitabine and interferon or zidovudine, along with the patient’s advanced age (75 years) and ECOG performance status of 2, systemic ATLL-directed therapy was deferred in favor of close clinical monitoring of the skin lesions.

The patient has continued regular oncologic follow-up for both her colorectal cancer and cutaneous ATLL. At the time of manuscript submission, she had completed three cycles of capecitabine with favorable tolerance. Notably, the zygomatic skin lesion demonstrated marked regression during this period, and the patient remained asymptomatic. Comprehensive clinicopathologic data for both malignancies are presented in Supplementary File 2.

### Systematic review

#### Study identification

**Fig. 3:**
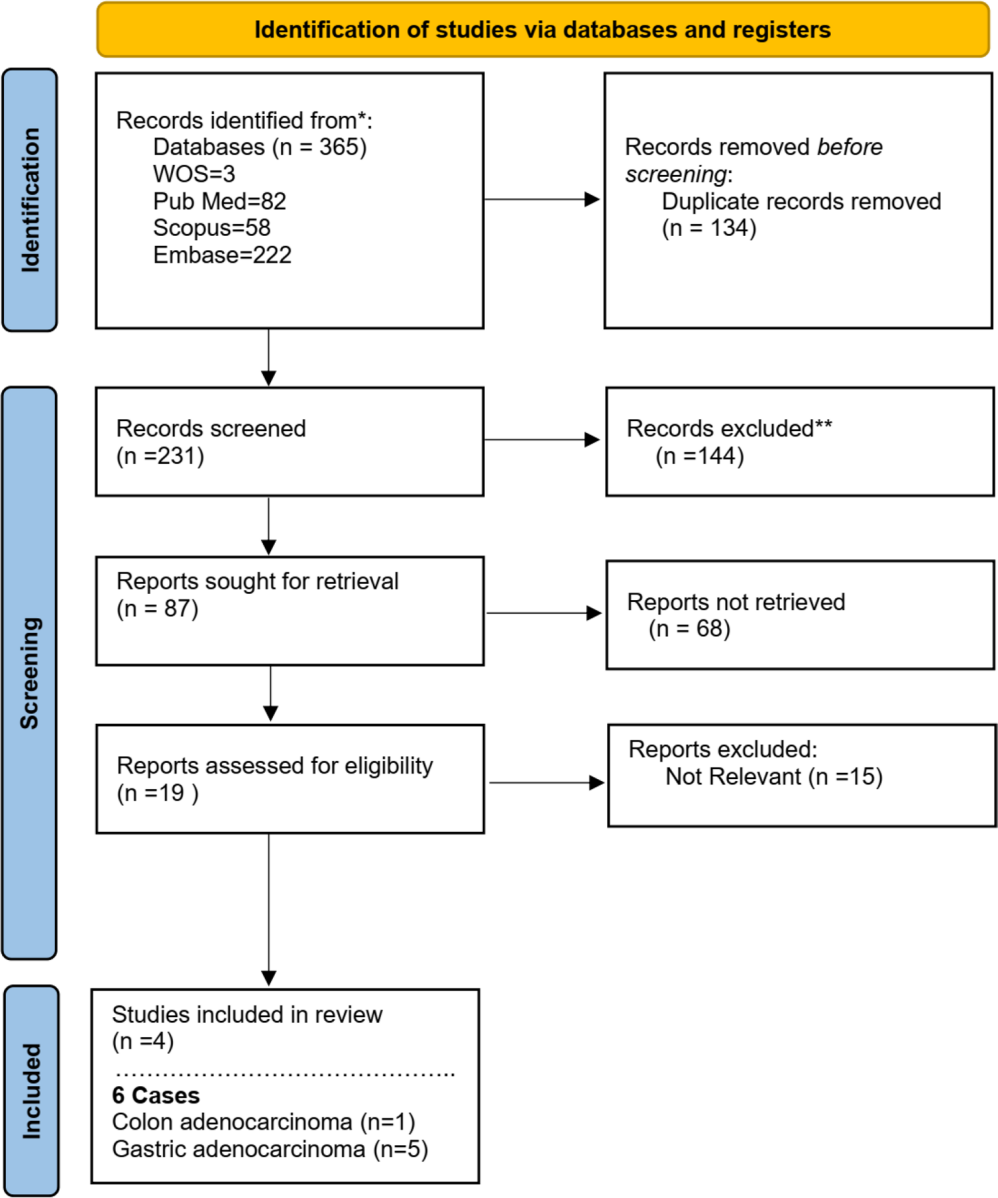
Study flowchart

The general structure of the predefined keywords, as well as the complete PRISMA flow diagrams with the resulting number of studies for each screening step is depicted in Fig. 3. According to our research strategy, a total of 365 published papers were identified across PubMed, Scopus, Embase and Web of Science. After removing 134 duplicates using End Note and Rayyan, 231 titles and abstracts were independently screened by two reviewers, resulting in the exclusion of 227 studies due to reasons such as irrelevancy, mislabeled populations, and language barriers. The remaining 6 articles met the inclusion criteria and underwent full-text assessment by two reviewers. All studies that met the inclusion criteria were available in English, either via open access or institutional subscriptions.

Two reviewers (HH and AZ) independently assessed the methodological quality of the included studies, resolving disagreements through discussion or consultation with a third reviewer (ZS) when necessary. Methodological quality assessment for case control studies is described in Supplementary File 3.

#### Characteristics of included studies

The studies included in this review describe six male patients with concurrent Adult T-cell Leukemia/Lymphoma (ATL) and gastrointestinal malignancies, all from Japan (Table 1). The patients’ ages ranged from 50 to 79 years. Common complaints included lymphadenopathy, abdominal symptoms such as nausea and epigastralgia, and systemic symptoms like weight loss and fever. A variety of gastrointestinal cancers were observed, including gastric adenocarcinoma (*n* = 5) [9–12] and colon adenocarcinoma (*n* = 1) [10]. Immunohistochemical markers for ATL were evaluated in all cases, with CD2, CD3, CD4, and CD8 being the most frequently tested markers. The levels of these markers varied, with CD2 showing the highest positivity in most cases. In addition to ATL markers, HTLV-1 proviral DNA and anti-HTLV-1 antibodies (ATLA) were detected in all patients, confirming the association of ATL with HTLV-1 infection. These studies highlight the complexity and variability in the clinical presentation, diagnosis, and outcomes of ATL in association with gastrointestinal cancers. The diagnostic markers and viral markers used in these cases are crucial for understanding the relationship between HTLV-1 infection and the development of both ATL and gastrointestinal malignancies.

**Table 1:** Clinicopathologic characteristics of case reports of HTLV-1–associated T-Cell Leukemia/Lymphoma (ATLL) coexisting with gastrointestinal cancers

Age (yr)/Sex	Complaint	Country/Race	Diagnosis	IHC Markers	Viral marker	Outcome/Follow-up	Ref.
64/M	Proteinuria, hypergammaglobulinemia, bilateral enlargement of several cervical, axillary and inguinal lymph nodes	Japan	smoldering ATL, B-Cell Lymphoma and early stage gastric adenocarcinoma	CD2 (85.5%), CD3 (63.4%), CD4 (47.1%), CD8 (48.6%), CD19 (3.5%), CD20 (2.4%), CD25 (36.8%), and CD45RO (62.7%)	HTLV-I pro-viral DNA and anti-HTLV-I Ab	no recurrence in gastric cancer	Kodama et al. 1995 [9]
76/M	Systemic lymph node swelling	Japan	ATL and moderately differentiated gastric adenocarcinoma	CD2 (71%), CD3 (20%), CD4 (46%), and CD8 (7%)	ATLA antibody and HTLV-I pro-viral DNA	-	Ono et al. 1989 [10]
79/M	Mass at his lower abdomen	Japan	ATL and well differentiated colon adenocarcinoma	CD2 (94%), CD3 (26%), CD4 (76%), and CD8 (15%)	ATLA antibody and HTLV-I pro-viral DNA	-	Ono et al. 1989 [10]
77/M	Lymph node swelling in the left axillary region	Japan	ATL and well differentiated gastric adenocarcinoma	CD2 (91%), CD3 (56%), CD4 (56%), and CD8 (18%)	ATLA antibody and HTLV-I pro-viral DNA	-	Ono et al. 1989 [10]
67/M	Nausea, epigastralgia and epigastrial tenderness	Japan	Smoldering ATL and advanced gastric carcinoma	CD3 (84.9%), CD4 (63.2%), CD8 (15.4%), CD4/CD8 (4.10)	anti-HTLV-I Ab	Gastric cancer brain metastasis	Tani et al. 2001 [11]
50/M	Weight loss, fever, superficial lymphadenopathy and occipital mass	Japan	ATLL and moderately differentiated gastric adenocarcinoma	CD30 and CD4 positive	HTLV-I pro-viral DNA and anti-HTLV-I Ab	-	Daibata et al. 2003 [12]

## Discussion

Our case describes a 75-year-old woman from an HTLV-1–endemic region who presented with acute large-bowel obstruction due to a sigmoid colon adenocarcinoma and was subsequently found to have concurrent cutaneous adult T-cell leukemia/lymphoma (ATLL) manifesting as erythematous facial plaques and a zygomatic nodule. Histopathology and immunophenotyping of the skin lesion revealed a mature CD3^+^/CD4^+^/CD25^+^ T-cell population with loss of CD7 and high Ki-67 in the setting of positive HTLV-1 serology, confirming cutaneous ATLL distinct from the pMMR colorectal adenocarcinoma.

The IHC panel in this case was selected to confirm a mature helper-T-cell phenotype and to distinguish ATLL from other T-cell lymphomas with overlapping features. Strong expression of CD2, CD3, CD4, and CD5 established that the infiltrate was composed of mature peripheral T cells. CD25 positivity—reflecting IL-2 receptor α-chain overexpression—is a classic feature of HTLV-1–driven neoplastic transformation and strongly supports ATLL [13, 14]. The loss of CD7, a frequent aberrancy in ATLL, helped separate this neoplasm from reactive T-cell infiltrates and many other CTCLs that typically retain CD7. The absence of CD8 excluded cytotoxic T-cell lymphomas, while CD20 negativity (if performed) would exclude B-cell neoplasms. CD30 negativity reduced the likelihood of primary cutaneous anaplastic large-cell lymphoma and large-cell transformation of mycosis fungoides. CCR4 negativity, although ATLL is often CCR4-positive, helped rule out other CCR4-expressing CTCLs and highlighted the immunophenotypic variability of cutaneous ATLL. Finally, ALK-1 negativity excluded ALK-positive anaplastic large-cell lymphoma. Overall, the combination of a CD4^+^/CD25^+^ mature T-cell phenotype with CD7 loss and absence of cytotoxic, B-cell, and ALK-related markers is most consistent with ATLL in the appropriate clinical and virologic setting [14].

In contrast to our study Yamada et al. examined 180 primary CRC resections from an HTLV-1–endemic region and identified 35 HTLV-1 carriers among them. CRC patients who were HTLV-1–positive were significantly older than non-carriers. Importantly, HTLV-1 carriers showed a remarkably lower rate of lymph-node metastasis and consequently lower overall tumour stage. Although relapse was less frequent in carriers, the trend did not reach significance. Tumour microenvironment analysis revealed a significantly higher density of FOXP3^+^ regulatory T cells (Tregs) in HTLV-1 carriers, and in situ hybridisation demonstrated HTLV-1–infected cells (HBZ-positive lymphocytes) clustered within the tumor stroma. Overall, the findings suggest that HTLV-1 infection suppresses lymph-node metastasis in CRC, potentially due to altered or dysfunctional Tregs and enhanced anti-tumor immune responses in HTLV-1–positive individuals [15]. Also, a case–control study (2009–2012) evaluated 265 Iranian patients with pathologically confirmed GC or CRC and 244 healthy controls with normal endoscopy/colonoscopy to assess any association between HTLV-1 infection and gastrointestinal cancers. Anti–HTLV-1 antibodies were measured using third-generation ELISA. The study included 201 GC patients, 64 CRC patients, and 244 controls, with similar mean ages across groups. HTLV-1 seropositivity was extremely low, detected in only 1 GC patient, no CRC patients, and 4 controls. Statistical analysis showed no significant association between HTLV-1 antibody positivity and either gastric or colorectal cancer. Overall, the findings indicate that HTLV-1 infection was not more common in GC or CRC patients and was actually detected less frequently in cancer patients than in healthy controls. Larger studies are recommended to clarify any potential relationship [16]. Another case–control study (2008–2011) evaluated 201 GC patients and 219 controls to investigate any association between HTLV-1 infection and GC. HTLV-1 antibodies were screened using ELISA and confirmed with Western blot. Most gastric tumors were located in the distal, non-cardia region, and the mean ages of patients and controls were similar. HTLV-1 seropositivity was rare in both groups, detected in only 0.5% of GC patients compared with 1.89% of controls, a pattern similar to reports from Japan, which suggest no positive association between HTLV-1 and gastric cancer risk. They conclude that the frequency of HTLV-1 infection was lower in GC patients than in controls, and recommend larger studies to clarify whether any relationship exists between HTLV-1 infection and gastric cancer in Iran [17].

Regulatory T (Treg) cells are enriched in CRC lesions and act to inhibit anti-tumor immune responses, thereby supporting tumor growth and progression [18]. It was demonstrated a significantly elevated density of FOXP3^+^ regulatory T cells in HTLV-1 carriers. Additionally, in situ hybridization detected HTLV-1 basic leucine zipper factor (HBZ)–positive cells, likely corresponding to lymphocytes localized within the stromal regions surrounding the cancer nests [15]. HTLV-1 infection can result in adult T-cell leukemia/lymphoma after a prolonged latency and is also responsible for several inflammatory disorders, such as HTLV-1–associated myelopathy (HAM) and HTLV-1–associated uveitis (HAU). The virus predominantly infects CD4^+^, CD25^+^, and FOXP3^+^ T cells. FOXP3 serves as the central transcription factor of regulatory T cells (Tregs), which characteristically express CTLA-4 and a high-affinity IL-2 receptor containing CD25. Through CTLA-4–mediated downregulation of CD80/CD86 on antigen-presenting cells and consumption of IL-2 via their high-affinity IL-2 receptor, Tregs suppress T-cell activation and induce cytokine deprivation–driven apoptosis of effector T cells [15, 19–21]. T-cell activation requires the simultaneous recognition of a specific major histocompatibility complex (MHC)–peptide complex by the T-cell receptor (TCR) and of B7-1 (CD80) or B7-2 (CD86) by the co-stimulatory receptor CD28. This dual engagement triggers T-cell proliferation, differentiation, and cytokine production, driving an effective immune response. In the absence of CD28-mediated co-stimulation, T cells either undergo apoptosis or enter a state of anergy. Following activation, upregulation of cytotoxic T-lymphocyte antigen 4 (CTLA-4; CD152) on T cells allows coligation of the TCR and CTLA-4, which induces cell-cycle arrest and terminates further T-cell activation, thereby providing a critical checkpoint in immune regulation [22].

IL-10 acts as an immunosuppressive cytokine that limits antitumor immunity in CRC liver metastases. It inhibits the proliferation and cytotoxic activity of both endogenous CD8+ T cells and CEA-specific CAR-T cells, and suppresses antigen-presenting cell function, including macrophage activation and MHC-I/II–mediated tumor antigen presentation. Blocking IL-10 enhances T cell infiltration and activation, increases CAR-T cell–mediated cytotoxicity, and significantly boosts cancer cell apoptosis, indicating that IL-10 normally restrains effective antitumor immune responses in in CRC liver metastases [23]. Also, IL-10 in CRC promotes tumor progression by mediating crosstalk between cancer cells and M2 macrophages. Specifically, IL-10 produced by CRC cells drives M2 macrophage polarization, which in turn enhances CRC cell migration and invasion. Migration and invasion inhibitory protein (MIIP) suppresses IL-10 production via the STING–TRAF3–NFκB2 pathway, limiting M2 macrophage polarization and tumor-promoting effects [24]. Notably, in the context of HTLV-1 infection, viral proteins such as HBZ and Tax can also stimulate IL-10 production, further enhancing immunosuppression. Overall, IL-10 in the colorectal tumor microenvironment suppresses anti-tumor immunity, inhibits T cell and dendritic cell function, promotes Treg and M2 macrophage activity, and, in the presence of HTLV-1, can be further upregulated by viral proteins, collectively facilitating tumor progression [25].

### Limitations

Despite the significant findings in our case, several limitations need to be mentioned. First, this study is based on a single patient, which limits the generalizability of the results. Larger cohort studies are essential to confirm the prevalence and clinical significance of concurrent sigmoid colon adenocarcinoma and cutaneous ATLL in HTLV-1–endemic regions. Our systematic review also identified only a small number of cases of HTLV-1-associated gastrointestinal malignancies, underscoring the rarity of such co-occurrences. Additionally, while immunohistochemistry provided valuable insights into the T-cell phenotype in our patient, further molecular studies, including genetic sequencing, are needed to better understand the mechanisms behind the co-occurrence of these two malignancies. Another limitation is the lack of long-term follow-up data on treatment outcomes, which would be important to assess how concurrent HTLV-1 infection may influence the prognosis of both cancers. Future research should explore the potential role of HTLV-1 in modifying the tumor microenvironment and its impact on the immune response in colorectal and cutaneous malignancies. Moreover, deeper investigation into the molecular pathways linking HTLV-1 infection with the development of ATLL and other cancers is necessary to better understand the virus’s oncogenic potential, as highlighted by our review of the existing literature.

## Conclusion

This case report highlights the rare coexistence of sigmoid colon adenocarcinoma and cutaneous adult T-cell leukemia/lymphoma (ATLL) in a 75-year-old woman from an HTLV-1–endemic region. The patient, initially diagnosed with large-bowel obstruction, was later found to have smoldering ATLL based on skin biopsy and positive HTLV-1 serology. Immunohistochemistry confirmed ATLL, distinguishing it from other T-cell lymphomas. Additionally, our systematic review of the literature revealed a limited number of reported cases of concurrent ATLL and gastrointestinal malignancies in HTLV-1–infected individuals, with findings suggesting that HTLV-1 infection may influence tumor development and the immune microenvironment. This case underscores the diagnostic challenges and the need for further research to explore the potential link between HTLV-1 and gastrointestinal cancers, as well as its broader oncogenic role.

## Electronic supplementary material

Below is the link to the electronic supplementary material.

Supplementary Material 1

Supplementary Material 2

Supplementary Material 3

## Data Availability

All data presented in this study are available as the tables, figures, and supplementary files. Additional datasets are available from the corresponding author on reasonable request.

## Abbreviations

ATLL: Adult T-cell Leukemia/Lymphoma
CRC: Colorectal Cancer
CTLA-4: Cytotoxic T-lymphocyte Antigen 4
GC: Gastric Cancer
HBZ: HTLV-1 Basic Leucine Zipper Factor
HTLV-1: Human T-Cell Lymphotropic Virus Type 1
IHC: Immunohistochemistry
MHC: Major Histocompatibility Complex
pMMR: Mismatch Repair-Proficient
Tregs: Regulatory T Cells
