# Supplementary material for: Concurrent sigmoid colon adenocarcinoma presenting with cutaneous adult T-cell leukemia/lymphoma: a rare case report
Source: Infect Agent Cancer. 2026 Apr 11;21:40. doi: 10.1186/s13027-026-00755-6 (PMC13185279; doi:10.1186/s13027-026-00755-6)
Supplement: Supplementary file 1 — Supplementary Material 1 [file 13027_2026_755_MOESM1_ESM.docx]

| Table 1. Query searches performed in each database and corresponding search strategy. | | | |
| --- | --- | --- | --- |
| Database | **Types of Searches^*^** | **Search Strategy (Final Query)** | **Count** |
| PubMed | **1** | (  "HTLV-I Infections"[Mesh] OR  "HTLV-I"[Mesh] OR  "HTLV-1"[tiab] OR "HTLV-I"[tiab] OR  "Human T-cell Lymphotropic Virus Type 1"[tiab] OR  "Human T-lymphotropic virus type 1"[tiab] OR  "Human T-lymphotropic virus type I"[tiab] OR  "Human T-cell Lymphotropic Virus Type I"[tiab]  )  AND  (  "Digestive System Neoplasms"[Mesh] OR  "Gastrointestinal Neoplasms"[Mesh] OR  "gastrointestinal neoplasm*"[tiab] OR "gastrointestinal cancer"[tiab] OR "GI cancer"[tiab] OR  "GI neoplasm*"[tiab] OR "gastrointestinal malignanc*"[tiab] OR "GI malignanc*"[tiab] OR  "Gastrointestinal Stromal Tumors"[tiab] OR "gastrointestinal stromal tumor"[tiab] OR "GIST"[tiab] OR  "esophageal cancer"[tiab] OR "esophageal neoplasms"[tiab] OR "esophagus neoplasm*"[tiab] OR  "esophageal carcinoma"[tiab] OR  "stomach neoplasm*"[tiab] OR "gastric cancer"[tiab] OR "stomach cancer"[tiab] OR  "colorectal neoplasm*"[tiab] OR "colorectal cancer"[tiab] OR "colon cancer"[tiab] OR "rectal cancer"[tiab] OR  "liver neoplasm*"[tiab] OR "liver cancer"[tiab] OR "hepatocellular carcinoma"[tiab] OR "cholangiocarcinoma"[tiab] OR  "pancreatic neoplasm*"[tiab] OR "pancreatic cancer"[tiab] OR "anal cancer"[tiab]  )  AND  (  "Case Reports"[Publication Type] OR  "case report"[tiab] OR "case study"[tiab] OR "case series"[tiab] OR "case presentation"[tiab]  ) |  |
|  | **2** | (  "Adult T-Cell Leukemia-Lymphoma"[Mesh] OR  "Adult T cell leukemia lymphoma"[tiab] OR "Adult T-cell leukemia lymphoma"[tiab] OR  "Adult T-cell leukemia/lymphoma"[tiab] OR "Adult T cell leukemia/lymphoma"[tiab] OR  "Adult T-cell lymphoma leukemia"[tiab] OR "Adult T cell lymphoma leukemia"[tiab] OR  "Adult T cell leukaemia lymphoma"[tiab] OR "Adult T-cell leukaemia lymphoma"[tiab] OR  "Adult T-cell leukaemia/lymphoma"[tiab] OR "Adult T cell leukaemia/lymphoma"[tiab] OR  "Adult T-cell lymphoma leukaemia"[tiab] OR "Adult T cell lymphoma leukaemia"[tiab] OR  "ATLL"[tiab] OR "ATL"[tiab]  )  AND  (  "Digestive System"[Mesh] OR  gastrointestinal[tiab] OR GI[tiab] OR  esophag*[tiab] OR gastric[tiab] OR stomach[tiab] OR colorectal[tiab] OR colon[tiab] OR rectal[tiab] OR  liver[tiab] OR biliary[tiab] OR gallbladder[tiab] OR pancreas[tiab] OR duodenal[tiab] OR small-intestin*[tiab] OR anal[tiab]  )  AND  (  "Case Reports"[Publication Type] OR  "case report"[tiab] OR "case study"[tiab] OR "case series"[tiab] OR "case presentation"[tiab]  ) |  |
| Scopus | **1** | TITLE-ABS-KEY(  HTLV-1 OR HTLV-I OR  "Human T-cell Lymphotropic Virus Type 1" OR  "Human T-lymphotropic virus type 1"  )  AND  TITLE-ABS-KEY(  "gastrointestinal neoplasm*" OR "gastrointestinal cancer" OR "GI cancer" OR "GI neoplasm*" OR  "gastrointestinal malignanc*" OR "GI malignanc*" OR  "Gastrointestinal Stromal Tumors" OR "gastrointestinal stromal tumor" OR GIST OR  "esophageal cancer" OR "esophageal neoplasms" OR "esophagus neoplasm*" OR "esophageal carcinoma" OR  "stomach cancer" OR "gastric cancer" OR "stomach neoplasm*" OR "gastric neoplasm*" OR  "colorectal cancer" OR "colon cancer" OR "rectal cancer" OR  "liver cancer" OR "hepatocellular carcinoma" OR "cholangiocarcinoma" OR "pancreatic cancer"  )  AND  TITLE-ABS-KEY("case report" OR "case series" OR "case study" OR "case presentation") |  |
|  | **2** | TITLE-ABS-KEY(  "Adult T-cell leukemia lymphoma" OR "Adult T cell leukemia lymphoma" OR  "Adult T-cell leukemia/lymphoma" OR "Adult T cell leukemia/lymphoma" OR  "Adult T-cell lymphoma leukemia" OR "Adult T cell lymphoma leukemia" OR  "Adult T-cell leukaemia lymphoma" OR "Adult T cell leukaemia lymphoma" OR  "Adult T-cell leukaemia/lymphoma" OR "Adult T cell leukaemia/lymphoma" OR  ATLL OR ATL  )  AND  TITLE-ABS-KEY(  gastrointestinal OR GI OR esophag* OR gastric OR stomach OR colorectal OR colon OR rectal OR  liver OR biliary OR gallbladder OR pancreas OR duodenal OR small-intestin* OR anal OR GIST  )  AND  TITLE-ABS-KEY("case report" OR "case series" OR "case study" OR "case presentation") |  |
| Web of Science (WOS) | **1** | TS=(  HTLV-1 OR HTLV-I OR  "Human T-cell Lymphotropic Virus Type 1" OR "Human T-lymphotropic virus type 1"  )  AND  TS=(  "gastrointestinal neoplasm*" OR "gastrointestinal cancer" OR "GI cancer" OR "GI neoplasm*" OR  "gastrointestinal malignanc*" OR "gastrointestinal stromal tumor*" OR GIST OR  "esophageal cancer" OR "esophageal neoplasm*" OR "esophageal carcinoma" OR  "stomach cancer" OR "gastric cancer" OR "stomach neoplasm*" OR  "colorectal cancer" OR "colon cancer" OR "rectal cancer" OR  "liver cancer" OR "hepatocellular carcinoma" OR "cholangiocarcinoma" OR  "pancreatic cancer"  )  AND  TS=("case report" OR "case series" OR "case study" OR "case presentation") |  |
|  | **2** | TS=(  "Adult T-cell leukemia/lymphoma" OR "Adult T cell leukemia/lymphoma" OR  "Adult T-cell leukemia lymphoma" OR "Adult T cell leukemia lymphoma" OR  "Adult T-cell lymphoma leukemia" OR "Adult T cell lymphoma leukemia" OR  "Adult T-cell leukaemia lymphoma" OR "Adult T cell leukaemia lymphoma" OR  "Adult T-cell leukaemia/lymphoma" OR "Adult T cell leukaemia/lymphoma" OR  ATLL OR ATL  )  AND  TS=(  gastrointestinal OR GI OR esophag* OR gastric OR stomach OR colorectal OR colon OR rectal OR  liver OR biliary OR gallbladder OR pancreas OR duodenal OR small-intestin* OR anal OR GIST  )  AND  TS=("case report" OR "case series" OR "case study" OR "case presentation") |  |
| Embase | **1** | ('human t lymphotropic virus 1 infection'/exp OR 'human t lymphotropic virus 1'/exp  OR HTLV-1:ti,ab,kw OR HTLV-I:ti,ab,kw  OR 'human t-lymphotropic virus type 1':ti,ab,kw)  AND  ('digestive system cancer'/exp OR 'gastrointestinal cancer'/exp OR  'esophageal cancer'/exp OR 'stomach cancer'/exp OR 'colorectal cancer'/exp OR  'liver cancer'/exp OR 'hepatocellular carcinoma'/exp OR 'cholangiocarcinoma'/exp OR  'pancreatic cancer'/exp OR 'gastrointestinal stromal tumor'/exp  OR "gastrointestinal neoplasm*":ti,ab,kw OR "GI cancer":ti,ab,kw)  AND  ('case report'/exp OR 'case report':ti,ab,kw OR 'case series':ti,ab,kw  OR 'case study':ti,ab,kw OR 'case presentation':ti,ab,kw) |  |
|  | **2** | ('adult t cell leukemia/lymphoma'/exp OR 'adult t-cell leukemia/lymphoma':ti,ab,kw OR ATLL:ti,ab,kw OR ATL:ti,ab,kw)  AND  (gastrointestinal:ti,ab,kw OR GI:ti,ab,kw OR esophag*:ti,ab,kw OR gastric:ti,ab,kw OR stomach:ti,ab,kw OR  colorectal:ti,ab,kw OR colon:ti,ab,kw OR rectal:ti,ab,kw OR liver:ti,ab,kw OR biliary:ti,ab,kw OR gallbladder:ti,ab,kw OR  pancreas:ti,ab,kw OR duodenal:ti,ab,kw OR small-intestin*:ti,ab,kw OR anal:ti,ab,kw OR GIST:ti,ab,kw)  AND  ('case report'/exp OR 'case report':ti,ab,kw OR 'case series':ti,ab,kw OR 'case study':ti,ab,kw OR 'case presentation':ti,ab,kw) |  |
| Search 1: HTLV-1 infection + GI cancer + Case reports  Search 2: ATLL (Adult T-cell Leukemia/Lymphoma) + GI involvement + Case reports | | | |
