# Supplementary material for: Concurrent sigmoid colon adenocarcinoma presenting with cutaneous adult T-cell leukemia/lymphoma: a rare case report
Source: Infect Agent Cancer. 2026 Apr 11;21:40. doi: 10.1186/s13027-026-00755-6 (PMC13185279; doi:10.1186/s13027-026-00755-6)
Supplement: Supplementary file 2 — Supplementary Material 2 [file 13027_2026_755_MOESM2_ESM.docx]

| Supplementary Table 1. Clinicopathologic data Concurrent Sigmoid Colon Adenocarcinoma Presenting with Cutaneous Adult T-Cell Leukemia/Lymphoma: A Rare Case Report. | | |
| --- | --- | --- |
| Category | **Parameter** | **Finding** |
| Colon Adenocarcinoma | Tumor site | Descending/Sigmoid colon |
|  | Histologic type | Adenocarcinoma |
|  | Histologic grade | Moderately differentiated |
|  | Tumor size | 7 × 3 × 1 cm |
|  | Lymphovascular invasion | Absent |
|  | Perineural invasion | Absent |
|  | Tumor budding | Intermediate (5 buds) |
|  | Margins | Negative |
|  | Lymph nodes | 0/10 involved |
|  | TNM classification | pT3N1c* |
| Cutaneous ATLL – Immunophenotype | CD3 | Positive |
|  | CD4 | Positive |
|  | CD25 | Positive |
|  | Ki-67 | High |
|  | CD7 | Negative/Lost |
|  | CD8 | Negative |
|  | CD20 | Negative |
|  | CD30 | Negative |
|  | CD56 | Negative |
|  | ALK-1 | Negative |
